# Evaluation of Antidiabetic and Antioxidant Activities of Fruit Pulp Extracts of *Cucurbita moschata* Duchesne and *Cucurbita maxima* Duchesne

**DOI:** 10.1155/2023/1124606

**Published:** 2023-06-22

**Authors:** Apinya Suwannapong, Chusri Talubmook, Wilawan Promprom

**Affiliations:** Department of Biology, Faculty of Science, Mahasarakham University, Maha Sarakham 44150, Thailand

## Abstract

**Objective:**

To evaluate and compare the antidiabetic and antioxidant activities of fruit pulp extracts from *Cucurbita moschata* (PCMOS) and *Cucurbita maxima* (PCMAX).

**Methods:**

The antidiabetic activity was carried out in vivo by orally and daily giving the extracts at a dose of 500 mg/kg·b.w. to the streptozotocin-induced diabetic male albino Wistar rats for six weeks. After the period of administration, blood glucose levels, body weight, serum insulin, morphology of islets of Langerhans, biochemical parameters, and haematological values of the rats were determined. Meanwhile, the antioxidant activity was carried out in vitro by determination of total phenolic and flavonoid contents, DPPH radical scavenging activity, and ferric reducing antioxidant power.

**Results:**

PCMAX significantly (*p* < 0.05) reduced blood glucose levels but increased the body weight, serum insulin levels, size and number of islets of Langerhans, and *β*-cell number of the treated diabetic rats more than PCMOS did. However, they did not alter biochemical parameters and haematological values of the treated diabetic rats. PCMAX possessed total phenolic and flavonoid contents and showed DPPH scavenging and FRAP reducing antioxidant power more significantly (*p* < 0.05) than PCMOS.

**Conclusions:**

According to the obtained results, it is indicated that PCMOS and PCMAX possess antidiabetic and antioxidant activities. PCMAX possesses more potent antidiabetic and antioxidant activities than PCMOS. These are probably due to PCMAX providing polysaccharide and total phenolic and flavonoid contents more than PCMOS.

## 1. Introduction

Diabetes mellitus (DM) is a severe metabolic disease that significantly impacts the health, quality of life, and life expectancy of patients, as well as the healthcare system [1]. It is a chronic carbohydrate, fat, and protein metabolism disorder characterized by high blood glucose levels resulting from the absence of insulin or insulin resistance [2, 3]. DM can cause substantial morbidity, mortality, and long-term complications [4]. The prevalence of DM will increase to 370 million or more by 2030 [5]. There is an urgent need for therapeutic agents to treat DM with fewer side effects. Synthetic antidiabetic agents often possess serious side effects. As the availability of antidiabetic agents in developing countries is limited, alternative approaches for treating diabetes using suitable antidiabetic agents from food sources are therefore being examined. Food has been documented to have antidiabetic properties, including cucurbits in the Cucurbitaceae family [6]. Pumpkin, a vegetable, belongs to the Cucurbitaceae. This family comprises about 130 genera with 800 species and can be cultivated worldwide [7, 8]. Pumpkin has been used in traditional systems of medicine. It exhibits various pharmacological actions, such as antidiabetic, anticancer, antihypertension, antioxidant, anti-inflammation, antihyperlipidemic, antimicrobial, and immunomodulation [9]. Its fruits are a valuable part of the plant with a high nutritional value [10] and are a good source of polyphenols [11], vitamin A, iron, phosphorus, calcium, and polysaccharides. Most bioactive chemicals are concentrated in its fruits [12].

*C. moschata* is a potential source of many antioxidant effects, such as carotenoids and polyphenols. Its fruit pulp is rich in many essential human and animal body compounds, such as amino acids, carbohydrates, polysaccharides, vitamins, and minerals [13, 14]. Its fruits are also rich in polysaccharides that show strong hypoglycemic and antidiabetic effects [15–18]. A previous investigation showed that pumpkin is reported to reduce blood glucose in type 2 diabetic patients [19].

*C*. *maxima* is traditionally used in Mexico, China, and India for its hypoglycemic effect helpful in controlling blood glucose [20, 21]. It has high potential for antidiabetic activity and can decrease high blood glucose levels in critically ill diabetic patients [21]. Its fruit juice, hydroalcoholic extract [20], fruit flesh powder [22], alcoholic seed extract [23], aqueous extract [24], and methanol extract [25, 26] significantly decreased hyperglycemia in STZ-induced diabetic rats. Studies in animal and human models revealed that treatment with some cucurbits exhibited hypoglycemic effects and stimulated *β*-cell regeneration. At least one of the bioactive components which produced these effects was a polysaccharide [18].

Crude polysaccharides from pumpkin showed hypoglycemic activity in alloxan-induced diabetic rats [27]. Moreover, the pumpkin polysaccharide extract gave excellent healing potential in alloxan-induced diabetic male ICR mice [28] and alloxan-induced diabetic rabbits [17]. It is thought that these effects may have been due to the antioxidant nature of the polysaccharide protecting pancreatic *β* cells [29], resulting in a reduced need for insulin in diabetic patients [30]. Pumpkin has been reported to be a source of antioxidants such as polyphenols and carotenoids [31], acidic polysaccharide extracted from pumpkin [32], water-soluble polysaccharide fraction from fruits of *C. maxima* [33], fractions of water-soluble polysaccharide from the fruit of pumpkin [29], methanol extract from aerial parts of *C. maxima* [25], mature fruits of *C. moschata* [34], methanolic and aqueous extracts of *C. moschata* fruits [35], methanolic extract of leaves of *C. maxima* [10], ethanol extracts of *C. moschata* and *C. maxima* [36], leaf extract of *C. moschata* [37], *C*. *moschata* flour [38], and flesh of *C. maxima* [39].

To elucidate the underlying mechanisms that could lead to the antidiabetic and antioxidant activities of the pumpkin, the present study was therefore undertaken to investigate the antidiabetic activity of extracts from the fruit pulp of *C. moschata* and *C. maxima* in STZ-induced diabetic rats and the antioxidant activity by DPPH scavenging and FRAP assays and evaluate the total phenolic and flavonoid contents. The results might help identify novel and relevant natural resources for the treatment of diabetes.

## 2. Materials and Methods

### 2.1. Plant Materials

Fresh mature fruits of *C. moschata* purchased from local gardens in Yasothon Province and *C. maxima* from a local market in Khon Kaen Province, Northeastern Thailand, were used in this study. The pumpkin fruit specimens were identified and authenticated by a botanist in the Department of Biology, Faculty of Science, Mahasarakham University, Thailand. Voucher plant specimens (MSU-SAMOS and MSU-SAMAX) have been deposited at the same department.

### 2.2. Preparation of Extracts

The plant fruits were washed, and the fruit pulp was cut into small pieces, dried in a hot air oven at 50°C. The dried samples were powdered using an electric grinder. The plant powder was extracted according to the procedure by Quanhong et al. [29] with slight modifications. The plant powder was separately suspended in distilled water at a ratio of 1 g powder:20 mL of distilled water, stirred in a water bath at 45°C for 16 h, and cooled and centrifuged at 4,800 rpm for 25 min. The supernatant was collected, concentrated to a quarter of the original volume by evaporating under reduced pressure at 45°C, and filtered to remove any residues. Three volumes of 95% ethanol were added to the filtrate to induce precipitation. The precipitated preparations were centrifuged at 4,800 rpm for 25 min. The pellet was collected, washed with ethanol, and freeze-dried. Each preparation was dissolved in distilled water (1 g/50 mL), dialysed against distilled water at 4°C for 72 h, and then freeze-dried. The yields of extracts from *C. moschata* (PCMOS) and *C. maxima* (PCMAX) were 1.15 and 1.10% DW, respectively. Because the active ingredient found in pumpkin has been reported to be polysaccharide [15], the PCMOS and PCMAX extracts were assayed for polysaccharides using the phenol-sulfuric acid method. A 2 mL aliquot of the extract suspension was mixed with 1 mL of 5% aqueous solution of phenol in a test tube. Five mL of concentrated sulfuric acid was added rapidly to the mixture. After allowing it to stand for 10 min, the mixture was mixed thoroughly using a vortex mixer for 30 s and placed for 20 min in a water bath at room temperature (27°C) for colour development. Light absorption at 490 nm was recorded with a spectrophotometer. The percentage polysaccharide in PCMOS and PCMAX was calculated and found to be 33.41 and 36.99%, respectively.

### 2.3. Determination of Antidiabetic Activity

#### 2.3.1. Experimental Animals

Male albino Wistar rats (200–250 g body weight) from the Laboratory Animal Centre, Suranaree University of Technology, Thailand, were used to assess antidiabetic activity. The rats were housed at room temperature (23 ± 2°C) and relative humidity (50–55%), and exposed to a 12 h day-night cycle, and fed with a standard commercial pellet diet and water *ad libitum*. Animals were maintained in accordance with the guidelines of the Committee Care and Use of Laboratory Animal Resource, National Research Council Thailand, and the advice of the Institutional Animal Care and Use Committee, Mahasarakham University, Thailand, with approval number IACUC/MSU 0020/2017.

#### 2.3.2. Induction of Diabetes

The overnight fasting rats were induced to be diabetic by a single intraperitoneal injection of 65 mg/kg·b.w. streptozotocin (STZ, Sigma Chemicals, St. Louis, MO), freshly prepared in 20 mM citrate buffer pH 4.5. After STZ injection, the rats were provided with 2% sucrose in drinking water for 48 h to alleviate discomfort after initiating the hypoglycemic phase. After 3 days of diabetic induction, fasting blood glucose (FBG) of each rat was assessed by using a glucometer (Accu-Check, Roche, Manheim, Germany). The rats with FBG at or above 126 mg/dL were considered to be diabetic and employed in the study [40].

#### 2.3.3. Experimental Design

A total of 30 rats, 6 normal rats and 24 diabetic rats, were used. The rats were divided into five groups with six rats in each. Group I: normal rats were treated with 0.5% Tween 80 **(**normal controls). Group II: diabetic rats were treated with 0.5% Tween 80 (diabetic controls). Groups III, IV, and V: diabetic rats were treated with PCMOS 500 mg/kg·b.w., PCMAX 500 mg/kg·b.w., and glibenclamide 0.5 mg/kg·b.w., respectively. PCMOS and PCMAX were suspended in 0.5% Tween 80 and administered orally to the rats using an orogastric tube. The volume of administration was 10 mL/kg·b.w [41]. The oral administration of PCMOS, PCMAX, and the standard drug, glibenclamide, was carried out once a day for a period of six weeks. Body weight, fasting blood glucose, haematological values, serum insulin levels, biochemical parameters for lipid profiles, renal and hepatic functions, and pancreatic histological observation of the rats were determined.

### 2.4. Determination of Body Weight and Fasting Blood Glucose

Before the commencement of the experiments, body weight and fasting blood glucose levels of all rats were monitored. After the administration of PCMOS, PCMAX, and glibenclamide, the body weight and fasting blood glucose levels were measured weekly for 6 weeks. The fasting blood glucose level was evaluated from a drop of fasting blood drawn by the tail prick method using a glucometer (Accu-Check, Roche, Manheim, Germany).

### 2.5. Determination of Haematological Values

At the end of the experiments, the rats were made to fast overnight and were sacrificed under ether anesthesia. Blood samples were collected from the rat hearts by cardiac puncture and heparinized. The heparinized blood was used for the determination of haematological values, including white blood cells (WBCs), red blood cells (RBCs), hemoglobin (Hb), hematocrit (Hct), neutrophils (Neu), lymphocytes (Lym), monocytes (Mono), and platelets (Plt) by using an automatic blood chemical analyzer (BT 2000 plus, Germany).

### 2.6. Determination of Serum Insulin Levels

The blood sample collected from each rat heart was centrifuged to separate blood serum. The insulin levels in the serum were determined using a radio immune assay kit (MP Biomedicals; Orangeburg, NY, USA) and detected using an automatic gamma counter (Wallac 1470 Wizard; Perkin Elmer Instrument; Überlingen, Germany).

### 2.7. Estimation of Biochemical Parameters

The biochemical parameters in the rat serum, including total cholesterol, triglycerides, HDL cholesterol, LDL cholesterol, total protein, blood urea nitrogen (BUN), creatinine (Crea), aspartate aminotransferase (AST), alanine aminotransferase (ALT), and alkaline phosphatase (ALP), were estimated following enzyme colorimetric methods using automate SYNCHRON LX20 PRO.

### 2.8. Histological Observation

After scarification, the rat pancreas was removed immediately and prepared for histological observation. The pancreas was washed with normal saline, fixed in 10% formaldehyde, embedded in paraffin, and sectioned using a rotary microtome. Slides of the sections of approximately 5 microns were routinely stained with hematoxylin and eosin (H&E) and were qualitatively (morphological) and quantitatively (morphometric) analyzed according to Berraaouan et al. [42] with some modifications.

### 2.9. Determination of Antioxidant Activity

The antioxidant activity of PCMOS and PCMAX was determined by using the 2, 2- diphenyl-1-picrylhydrazyl (DPPH) free radical scavenging assay and ferric reducing antioxidant power (FRAP) assay. As antioxidant potential of many plants is mainly due to phenolic components, total phenolic and flavonoid contents in PCMOS and PCMAX were also investigated.

### 2.10. DPPH Free Radical Scavenging Assay

The antioxidant activity of PCMOS and PCMAX using the DPPH assay was assessed according to the method by Nakornriab and Krasaetep [43] with suitable modifications. PCMOS and ECMAX at the concentrations of 0.1, 0.2, 0.4, 0.6, 0.8, and 1 mg/mL were mixed with 3.0 mL of 0.1 mM DPPH solution. The mixture was shaken and incubated at 37°C for 30 min in the dark. Absorbance at 517 nm was recorded using a spectrophotometer. Ascorbic acid was used as a reference reagent. The DPPH radical scavenging assay was carried out with three replicates. Percentage inhibition of DPPH radicals was calculated using the following equation:

(1) $$\begin{array}{c} \%\;inhibition=\left[1-\left(\frac{absorbance\;of\;the\;solution\;with\;extracts}{absorbance\;of\;the\;solution\;without\;extracts}\right)\right]\times 100\text{.} \end{array}$$

IC 50 of PCMOS, PCMAX, and ascorbic acid was estimated from plots of % inhibition vs. concentration.

### 2.11. Ferric Reducing Antioxidant Power (FRAP) Assay

The FRAP assay was performed using a modified method described by Zolfaghari et al. [44]. PCMOS or PCMAX solution (100 *µ*L of 10 mg/mL) was mixed with 3 mL of FRAP solution (300 mM acetate buffer, pH 3.6, 10 mM/L TPTZ in 40 mM/L HCl, and 20 mM/L FeCl_3_ anhydrous). The working FRAP reagent was prepared freshly by mixing acetate buffer, TPTZ solution, and FeCl_3_ anhydrous in the ratio of 10 : 1 : 1. The prepared FRAP solution was incubated at 37°C for 4 min. The absorbance was then measured at 593 nm using a UV-visible spectrophotometer. The results were expressed as millimolar ferrous sulphate per gram of dry weight (mM Fe (II)/g DW).

### 2.12. Determination of Total Phenolic Content

The total phenolic content in PCMOS and PCMAX was determined calorimetrically using the Folin–Ciocalteu reagent according to the modified method by Bonoli et al. [45]. 50 *µ*L of the extracts (10 mg/mL) was mixed with 1.5 mL of the 10% Folin–Ciocalteu reagent (diluted 10 fold with distilled water). The mixed solution was incubated at room temperature for 15 min, and then, 1.5 mL of 10% (w/v) sodium carbonate solution was added. The mixture was shaken and incubated again at room temperature for 15 min. Gallic acid was used as a standard agent. The absorbance of all samples was measured at 750 nm using a UV-visible spectrophotometer. Total phenolic content was calculated by comparison to a gallic acid standard curve and expressed as mg gallic acid equivalent/g (mg GAE)/g.

### 2.13. Determination of Total Flavonoid Content

The amount of total flavonoid content of PCMOS and PCMAX was determined according to the modified method by Prasad et al. [46]. PCMOS or PCMAX (250 *µ*L of 10 mg/mL) was mixed with 1.25 mL of deionized water and 75 *µ*L of 5% sodium nitrite (NaNO_2_) solution and allowed to stand for 5 min at room temperature. Aluminium chloride (AlCl_3_) (150 *µ*L of 10% solution) was added to the mixture and allowed to react for 6 min at room temperature. Then, 500 *µ*L of 1 M sodium hydroxide (NaOH) and 775 *µ*L of distilled water were added to the mixture. The absorbance of all samples was immediately measured at 510 nm using a UV-visible spectrophotometer. The samples were assayed in triplicate. The amount of total flavonoid content was calculated using a catechin calibration curve and expressed in mgCE/g.

### 2.14. Data Analysis

Data were expressed as the mean ± standard error of mean (SEM). The significant difference in data between different groups was compared using one-way analysis of variance (ANOVA) followed by Duncan's test. *p* < 0.05 was considered to indicate a significant difference.

## 3. Results

### 3.1. Determination of Antidiabetic Activity

**Figure 1:**
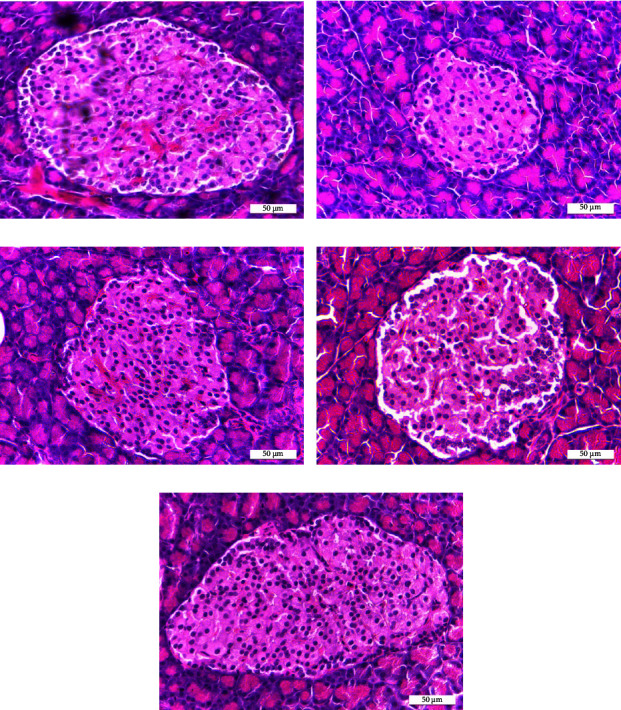
Representative light micrographs of islets of Langerhans in normal controls (a), diabetic controls (b), diabetic rats treated with PCMOS (c), diabetic rats treated with PCMAX (d), and diabetic rats treated with glibenclamide (e).

The results of body weight, haematological values, blood glucose levels, serum insulin levels, number of *β* cells, size of islets of Langerhans, biochemical parameters including lipid profiles, and renal and hepatic function parameters of normal controls, diabetic controls, and diabetic rats treated with PCMOS, PCMAX, and glibenclamide are presented in Tables 1–4 and Figure 1, respectively.

### 3.2. Body Weight

At the initial stage, the body weight of the rats was not different. However, at the end of the experiments, the body weight of the diabetic controls decreased significantly (*p* < 0.05) when compared to that of the normal controls (301.56 ± 11.12 vs. 361.33 ± 10.95 g). It increased significantly (*p* < 0.05) in the diabetic rats treated with PCMOS (354.16 ± 7.44 g) and PCMAX (359.66 ± 7.74 g) compared to that in the diabetic controls and in the diabetic rats treated with glibenclamide (354.16 ± 8.96 g).

### 3.3. Fasting Blood Glucose Levels

At the initial stage, fasting blood glucose of the diabetic rats was significantly higher (*p* < 0.05) than that of the normal controls. At the end of the experiments, the fasting blood glucose level of the diabetic controls (186.66 ± 15.70 mg/dL) increased significantly (*p* < 0.05) compared to that in the normal controls (83.07 ± 11.47 mg/dl). In contrast, it reduced significantly (*p* < 0.05) in the diabetic rats treated with PCMOS (102.16 ± 11.20 mg/dl) and PCMAX (96.00 ± 13.05 mg/dl) compared to that in the diabetic controls, but it was not different from that in the diabetic rats treated with glibenclamide (96.66 ± 12.32 mg/dL).

### 3.4. Haematological Values

The determination of haematological values revealed that white blood cells (WBCs), red blood cells (RBCs), hemoglobin (Hb), hematocrit (Hct), neutrophils (Neu), lymphocytes (Lym), and monocytes (Mono) of all groups of rats were not different. However, platelet counts of the diabetic controls (9.03 ± 1.12×10^3^ cells/mm^3^), diabetic rats treated with PCMOS (9.26 ± 1.23×10^3^ cells/mm^3^), and diabetic rats treated with glibenclamide (8.85 ± 1.51 × 10^3^ cells/mm^3^) increased slightly (7.6.45 ± 2.26 × 10^3^ cell/mm^3^) and increased significantly (*p* < 0.05) in the diabetic rats treated with PCMAX (9.69 ± 1.34 × 10^3^ cells/mm^3^) compared to those in the normal controls.

### 3.5. Serum Insulin Level

The serum insulin level of the diabetic controls (12.14 ± 0.34 *µ*IU/mL) decreased significantly (*p* < 0.05) compared with that of the normal controls (23.88 ± 1.38 *µ*IU/mL). In contrast, it increased significantly (*p* < 0.05) in the diabetic rats treated with PCMAX (16.64 ± 3.20 *µ*IU/mL) and increased slightly in the diabetic rats treated with PCMOS (13.00 ± 0.20 *µ*IU/mL). However, it was less than that in the diabetic rats treated with glibenclamide (24.80 ± 0.50 *µ*IU/ml) which was close to that in the normal controls.

### 3.6. Biochemical Parameters

The lipid profiles, including total cholesterol (TC), triglycerides (TGs), and low-density lipids (LDLs), increased significantly (*p* < 0.05), but high-density lipid (HDL) decreased significantly (*p* < 0.05) in the diabetic controls compared to those in the normal controls. On the other hand, TC, TG, and LDL decreased significantly (*p* < 0.05), while HDL increased significantly (*p* < 0.05) in the diabetic rats treated with PCMOS, PCMAX, and glibenclamide.

#### 3.6.1. Renal Function

TP in the diabetic controls decreased significantly (*p* < 0.05) compared to that in the normal controls. In contrast, it increased significantly (*p* < 0.05) in the diabetic rats treated with PCMOS, PCMAX, and glibenclamide and was similar to that in the normal controls. However, BUN and creatinine in the diabetic controls and diabetic rats treated with PCMOS, PCMAX, and glibenclamide were not different and were not different from those in the normal controls.

#### 3.6.2. Hepatic Function

AST in the diabetic controls increased significantly (*p* < 0.05) compared to that in the normal controls. However, it decreased significantly (*p* < 0.05) in the diabetic rats treated with PCMOS, PCMAX, and glibenclamide and was not different from that in the normal controls. Changes in ALT were not found among different groups of rats. ALP in the diabetic controls increased significantly (*p* < 0.05) but decreased significantly (*p* < 0.05) in the diabetic rats treated with PCMOS, PCMAX, and glibenclamide compared to that in the diabetic controls. Interestingly, ALP in the diabetic rats treated with PCMOS, PCMAX, and glibenclamide was comparable to that in the normal controls.

### 3.7. Histological Observation

Histological observations revealed that the pancreatic tissues of the diabetic controls experienced morphological changes with injury and death of pancreatic *β* cells leading to a decrease in the size of islets of Langerhans (143.74 ± 2.11*µ*; Figure 1(b)) and the number of *β* cells (84.06 ± 2.02 cells/unit; Table 3) compared with the normal controls (222.03 ± 2.71*µ*; Figure 1(a) and 190.37 ± 7.84 cells/unit; Table 3). Surprisingly, PCMOS and PCMAX attenuated diabetes-induced pancreatic morphological changes by significantly increasing (*p* < 0.05) the size of islets of Langerhans and the number of *β* cells in the diabetic rats treated with PCMOS (210.46 ± 4.05*µ*; Figure 1(c) and 134.02 ± 4.11 cells/unit; Table 3) and in the diabetic rats treated with PCMAX (214.27 ± 4.49*µ*; Figure 1(d) and 162.34 ± 3.72 cells/unit; Table 3). However, they were not different from those in the diabetic rats treated with glibenclamide (209.80 ± 3.46*µ*; Figure 1(e) and 158.25 ± 9.11 cells/unit).

### 3.8. Determination of Antioxidant Activity

The results of the determination of antioxidant activity of PCMOS and PCMAX are presented in Table 5. The DPPH assay revealed that PCMOS exhibited a scavenging DPPH radical less potent than PCMAX did with EC50 of 13.69 ± 0.45 vs. 8.03 ± 0.95 *µ*g/mL, respectively. However, PCMOS and PCMAX possessed a relatively low antioxidant activity compared to ascorbic acid (0.01 ± 0.02 *µ*g/mL); while using the FRAP assay, it was demonstrated that PCMOS exhibited less antioxidant activity by reducing Fe^2+^ than ECMAX did with 31.23 ± 3.15 vs. 42.67 ± 0.77 mMFe^2+^/g, respectively. PCMOS and PCMAX exhibited antioxidant activity as measured by the DPPH and FRAP assay in a concentration-dependent manner. PCMOS showed less TPC and TFC values than PCMAX with values of 5.27 ± 0.31 vs. 11.22 ± 0.19 mgGAE/g and 20.81 ± 0.09 vs. 55.08 ± 2.21 mgCE/g, respectively.

## 4. Discussion

Streptozotocin (STZ) is one of the most commonly used substances to induce diabetes in rats. STZ selectively destroys pancreatic insulin-secreting *β* cells causing diabetes similar to type 2 diabetes in humans according to [23], and induction of diabetes with STZ was associated with the characteristic loss of body weight due to increased muscle wasting and loss of tissue proteins because protein and fat are broken down to provide energy instead of blood glucose [47]; therefore, the body weight in the STZ-induced diabetic rats (diabetic controls) in the present study decreased significantly. The results are consistent with the report by Baldi et al. who found that a pumpkin concentrate caused a significant decrease in body weight in diabetic control rats [48]. Oral administration of PCMOS and PCMAX at a dose of 500 mg/kg to the rats once a day for six weeks increased the body weight of the PCMOS and PCMAX-treated diabetic rats because the characteristics of PCMOS and PCMAX were consistent with the pumpkin concentrate preparation, which increased the body weight of diabetic rats [23], and a water-soluble polysaccharide from pumpkin significantly increased the body weight of diabetic rabbits [17]. The action of PCMOS and PCMAX to increase the body weight of diabetic rats is probably due to their hypoglycemic property, which increases the utilization of blood glucose and, in turn, prevents the breakdown of fat and protein to provide energy.

STZ causes the destruction of the pancreatic insulin-secreting*β* cells, which reduces insulin and, in turn, results in a rise in blood glucose concentration, i.e., hyperglycemia [49]; the extracts, especially PCMAX, significantly decreased the blood glucose levels in the diabetic rats. This indicates that PCMAX contains a hypoglycemic agent which is responsible for lowering blood glucose levels. The blood glucose level of the diabetic control rats was still high during the experimentation for six weeks. However, the haematological values except for the platelet in the diabetic control rats and diabetic rats treated with PCMOS and PCMAX did not alter compared to those in the normal controls. This indicates that PCMOS and PCMAX produce nontoxicity as judged by haematological values. Serum insulin levels were found to be increased in the diabetic rats treated with PCMOS and PCMAX. This result is in agreement with results for the alcoholic seed extract of *C. maxima* which significantly reduced the elevated fasting blood glucose level by increasing the pancreatic secretion of insulin from *β* cells of islets of Langerhans in STZ-induced diabetic albino Wistar rats [23].

Lipid profiles, including total cholesterol (TC), triglycerides (TGs), and low-density lipoprotein (LDL), increased significantly. In contrast, the high-density lipoprotein (HDL) decreased significantly in the diabetic controls [50, 51]. In the present study, oral administration of PCMOS and PCMAX at a dose of 500 mg/kg to the diabetic rats once a day for six weeks significantly decreased TC, TG, and LDL levels but increased HDL levels in the treated diabetic rats. In agreement with the present data, there have been reports that the lipid profiles were changed to decrease total cholesterol, triglycerides, and LDL and increase HDL levels in the diabetic rats treated with pumpkin (*C. maxima*) at a dose of 400 mg/kg [52], and the administration of the pumpkin polysaccharide significantly lowered TC and TG in diabetic rabbits [17]. These results may be associated with the possible protective effect of the extract (mainly polysaccharides) on pancreatic *β* cells. In addition, these effects may be due to the low activity of cholesterol biosynthetic enzymes or the insignificant level of lipolysis under the control of insulin [53]. Moreover, in pumpkin seeds, unsaturated fatty acids, such as oleic acid and linoleic acid, can reduce cholesterol levels in rats [54]. These activities of PCMOS and PCMAX probably involve inhibiting the absorption of bile acids and cholesterol and enhancing the activity of LDL receptors [55].

PCMOS and PCMAX recovered TP and showed no effects on BUN as BUN in the diabetic controls, diabetic rats treated with PCMOS, PCMAX, and glibenclamide, and normal controls was not different. These results indicate the nontoxicity of PCMOS and PCMAX in renal function.

AST and ALP increased in the diabetic controls, but they decreased in the diabetic rats treated with PCMOS and PCMAX, and the levels were close to those in the normal controls. In addition, ALT in all rats was not different. These results indicate that PCMOS and PCMAX can improve the pathological hepatic function resulting from STZ-induced diabetes.

In the present study, histological observation revealed a difference in the mean diameter and the number of Langerhans islets and the number of pancreatic *β* cells between the diabetic controls and the normal controls. Decreasing the size of islets of Langerhans and the number of pancreatic *β* cells indicated the destruction of pancreatic tissues in diabetic rats. Aboulthana et al. reported that STZ caused inflammatory changes in pancreatic islets of diabetic rats. Consequently, this leads to atrophy in islands of the Langerhans cells associated with the vacillation of islet cells [56]. This might be due to the destruction of *β* cells and hence a decrease in pancreatic islets. This finding agrees with that of Sedigheh et al. who stated that histological analysis showed significant differences in the mean diameter and the number of Langerhans islets between the diabetic control group and the standard control group [57]. The mean diameter of islets in the diabetic group significantly decreased compared with that in the normal control group. Pancreatic *β* cells take up STZ *via* the GLUT 2 transporter, which leads to *β*-cell death by DNA fragmentation [58]. Due to the optical microscope images of islets of *β* cells of experimental animals, this suggests that the oral administration of PCMOS and PCMAX was potentially effective after STZ damage. The histopathological investigation showed that PCMOS and PCMAX could prevent pancreatic *β*-cell damage and increase the size and number of islets of Langerhans, leading to an increase in insulin levels. It has been reported that the fruit flesh powder of *C. maxima* significantly improved blood glucose and insulin levels and healed Langerhans islets by increasing the number and size of Langerhans islets in STZ-induced diabetic rats, indicating the effects of pumpkin powder on the repair and restoration of pancreatic tissues [22], and the methanol extract of aerial parts of *C maxima* altered the histological profiles of the pancreas of the STZ-induced diabetic albino Wistar rats to match those in the normal controls [25] more closely. The activity of PCMOS and PCMAX was in agreement with that of the methanol extract of *C*. *maxima* which attenuated diabetes-induced pancreatic injury in STZ-induced diabetic rats [26]. Administration of antioxidants to diabetic rats significantly increased the number of *β* cells [59]. Antioxidant compounds could increase the number of pancreatic *β* cells by enhancing the repair and restoration of these cells. Moreover, flavonoids could protect DNA from oxidative damage to lessen the problem in *β* cells [60–62].

According to these findings, (1) the phytochemical investigation of pumpkins reveals the presence of polysaccharides [15], (2) chemicals from pumpkin fruit pulp that are hypoglycemic include polysaccharides [63], (3) polysaccharides cause a significant decrease in the blood glucose of STZ-induced diabetic rats [64], (4) powdered pumpkin has hypoglycemic activity in alloxan-induced diabetic rats [58], which is due to polysaccharide components [15, 28, 58, 65], (5) protein-bound polysaccharides from the fruit of pumpkin (*C. moschata*) could decrease blood glucose levels [29], and (6) the polysaccharide is the main chemical in extracts, with 33.41% in PCMOS and 36.99% in PCMAX. These suggest that the hypoglycemic activity of PCMOS and PCMAX could be partly due to polysaccharide content. Previous studies using high-performance liquid chromatography (HPLC) analysis found the content of free sugars and polysaccharides in *C. maxima* fruits [66]. In addition, *C. moschata* polysaccharide possessed significant cytoprotective effects on diabetes and antioxidative activity [32], flavonoid extracted from Cucurbitaceae possessed hypoglycemic activity in diabetic rats [67], and oral administration of pumpkin seed extracts produced a significant antidiabetic effect in controlling the blood glucose level, possibly due to flavonoids [22]. Thus, the extracts were significantly antidiabetic, and the antioxidant effect correlates with the presence of the components mentioned.

Based on the presence of total phenolic (TPC) and total flavonoid (TFC) content in PCMOS and PCMAX, PCMOS and PCMAX exhibited significant antidiabetic activity, almost the same as glibenclamide, an antidiabetic drug; the mechanism of action on antidiabetic activity of PCMOS and PCMAX may be due to the polysaccharide, phenolic, and flavonoid content improving STZ-induced damage to pancreatic islets of Langerhans and stimulated serum insulin secretion from pancreatic *β* cells resulting in a reduction of blood glucose.

The DPPH scavenging and ferric reducing activity assay revealed the antioxidant activity of PCMOS and PCMAX, and PCMOS exhibited less potent antioxidant activity than PCMAX. However, they showed a relatively low activity compared to ascorbic acid, a reference agent. Because phenolic and flavonoid compounds are responsible for antioxidant activity in plants [68], the phenolic compound present in plants possesses hydroxyl groups with radical scavenging activity; antioxidants, in interaction with DPPH, either transfer an electron or a hydrogen atom to DPPH [69]. The methanol extract from aerial parts of *C. maxima* exhibits potent antioxidant activity with free radical scavenging activity equivalent to BHT, and the antioxidant property may be due to phenolic and flavonoid contents [25]. Therefore, the antioxidant activity of PCMOS and PCMAX in the present study was partly due to total phenolic and flavonoid contents. Nevertheless, the factors which involve the antioxidant effect of C. *moschata* showed high genetic variability for synthesising phenolic compounds, carotenoids, and antioxidant activity [70]. While the ABTS radical scavenging activity of pumpkin tended to increase according to the level of pumpkin leaf powder [71], the fruit acetone extract has an antioxidant effect of being higher in *C. maxima* lines than in *C. moschata* lines [14], and the antioxidant activity is more affected by the type of cultivar than by the pumpkin species [13]. Furthermore, numerous studies have shown that administering antioxidants to diabetic rats increases the number of *β* cells [42, 59]. Therefore, the pancreatic protective effect and its hypoglycemic properties should be attributable, in part, to the antioxidant activity of these two fruit pulps [22].

## 5. Conclusions

The fruit pulp extracts from *C. moschata* (PCMOS) and *C. maxima* (PCMAX) exhibit both antidiabetic activity in STZ-induced diabetic rats and antioxidant activity. The extracts display the antidiabetic activity by increasing the *β*-cell number and size of islets of Langerhans which lead to reducing blood glucose levels in the treated diabetic rats. They exert the antioxidant activity by DPPH scavenging and ferric reducing activity. PCMAX exhibits more potent activities than PCMOS which may result from PCMAX giving more polysaccharides and total phenolic and flavonoid contents. In the present study, a single dose (500 mg/kg·b.w.) of the extracts was used for the antidiabetic activity assessment. In further studies, various doses of the extracts should be employed to determine whether the activity is in a dose-dependent manner.

## Figures and Tables

**Table 1:** Body weight of normal controls, diabetic controls, diabetic rats treated with PCMOS, diabetic rats treated with PCMAX, and diabetic rats treated with glibenclamide.

Groups	Body weight (g)	
	Initial weight	Final weight
Normal controls	244.63 ± 7.84^a^	361.33 ± 10.95^b^
DM	236.10 ± 4.67^a^	301.56 ± 11.12^a^
DM + PCMOS	238.32 ± 7.18^a^	354.16 ± 7.44^b^
DM + PCMAX	240.10 ± 8.09^a^	359.66 ± 7.74^b^
DM + Glib	238.16 ± 9.15^a^	354.16 ± 8.96^b^

Values are expressed as means ± S.E.M, *n* = 6 rats in each group. There was no significant difference between means that have the same alphabetical superscript letter in the same column (*p* < 0.05). Statistical analysis was carried out using one-way ANOVA followed by Duncan's test. DM = diabetic rats; Glib = glibenclamide.

**Table 2:** Haematological values of normal controls, diabetic controls, diabetic rats treated with PCMOS, diabetic rats treated with PCMAX, and diabetic rats treated with glibenclamide.

Groups	Haematological values							
	RBC (×10^6^ cells/mm^3^)	WBC (×10^3^ cells/mm^3^)	Hb (g/dl)	Hct (%)	Neu (%)	Lym (%)	Mono (%)	Plt (×10^3^ cells/mm^3^)
Normal controls	9.51 ± 0.12^a^	7.65 ± 3.45^a^	16.83 ± 0.31^a^	50.28 ± 0.59^a^	11.33 ± 0.49^a^	82.33 ± 0.49^a^	4.83 ± 0.70^a^	7.6.45 ± 2.26^a^
DM	9.32 ± 0.19^a^	5.83 ± 1.38^a^	16.45 ± 0.91^a^	48.78 ± 1.03^a^	13.33 ± 1.22^a^	80.83 ± 1.24^a^	4.83 ± 0.60^a^	9.03 ± 1.12^ab^
DM + PCMOS	9.71 ± 0.15^a^	8.90 ± 2.22^a^	16.86 ± 0.67^a^	49.48 ± 1.17^a^	15.00 ± 1.93^a^	80.00 ± 2.69^a^	3.66 ± 0.84^a^	9.26 ± 1.23^ab^
DM + PCMAX	9.80 ± 0.17^a^	8.10 ± 2.31^a^	16.80 ± 0.51^a^	48.71 ± 0.83^a^	13.16 ± 1.79^a^	82.00 ± 1.59^a^	3.33 ± 0.80^a^	9.69 ± 1.34^b^
DM + Glib	9.80 ± 0.08^a^	6.90 ± 2.12^a^	16.80 ± 0.72^a^	48.58 ± 1.21^a^	10.50 ± 1.74^a^	85.16 ± 1.51^a^	3.33 ± 0.33^a^	8.85 ± 1.51^ab^

Values are expressed as means ± S.E.M, *n* = 6 rats in each group. There was a significant difference between means that have the different superscript letters in the same column (*p* < 0.05). Statistical analysis was carried out using one-way ANOVA followed by Duncan's test. DM = diabetic rats; Glib = glibenclamide; RBC = red blood cell; WBC = white blood cell; Hb = hemoglobin; Hct = hematocrit; Neu = neutrophil; Lym = lymphocyte; Mono = monocyte; Plt = platelet.

**Table 3:** Fasting blood glucose, serum insulin level, number of *β* cells, and size of islets of Langerhans from normal controls, diabetic controls, diabetic rats treated with PCMOS, diabetic rats treated with PCMAX, and diabetic rats treated with glibenclamide.

Parameters	Groups				
	Normal controls	DM	DM + PCMOS	DM + PCMAX	DM + Glib
FBG (mg/dl)	83.07 ± 11.47^a^	186.66 ± 15.70^c^	102.16 ± 11.20^b^	96.00 ± 13.05^b^	96.6612.32^b^
Serum insulin (*µ*IU/mL)	23.88 ± 1.38^c^	12.14 ± 0.34^a^	13.00 ± 0.20^a^	16.64 ± 3.20^b^	24.80 ± 0.50^c^
Number of *β* cells (cells/unit)	190.37 ± 7.84^d^	84.06 ± 2.02^a^	134.02 ± 4.11^b^	162.34 ± 3.72^c^	158.25 ± 9.11^c^
Size of islets (micron)	222.03 ± 2.71^bc^	143.74 ± 2.11^a^	210.46 ± 4.05^b^	214.27 ± 4.49^bc^	209.80 ± 3.46^b^

Values are expressed as means ± S.E.M, *n* = 6 rats in each group. There was a significant difference between means that have the different superscript letters in the same row (*p* < 0.05). Statistical analysis was carried out using one-way ANOVA followed by Duncan's test. DM = diabetic rats; Glib = glibenclamide; FBG = fasting blood glucose.

**Table 4:** Biochemical values for determination of lipid profiles, renal function, and hepatic function in normal controls, diabetic controls, diabetic rats treated with PCMOS, diabetic rats treated with PCMAX, and diabetic rats treated with glibenclamide.

Biochemical values	Normal controls	DM	DM + PCMOS	DM + PCMAX	DM + Glib
*Lipid profiles*					
CHO (mg/dl)	62.08 ± 3.5^a^	77.62 ± 5.03^b^	75.81 ± 5.01^b^	74.82 ± 2.34^ab^	69.65 ± 5.20^ab^
TG (mg/dl)	128.37 ± 4.52^bc^	121.33 ± 1.14^bc^	109.05 ± 2.09^a^	106.61 ± 1.53^a^	117.04 ± 2.24^bc^
LDL (mg/dl)	15.17 ± 2.16^a^	21.53 ± 1.62^b^	18.34 ± 1.67^ab^	17.83 ± 2.25^ab^	21.82 ± 3.07^b^
HDL (mg/dl)	60.86 ± 2.91^b^	49.52 ± 4.37^a^	56.57 ± 2.36^ab^	57.09 ± 3.22^ab^	53.64 ± 3.41^ab^

*Biochemical parameters*					
*Renal function*					
TP (g/dl**)**	6.66 ± 0.82^b^	5.61 ± 0.34^a^	6.75 ± 0.42^b^	6.48 ± 0.63^b^	6.07 ± 0.51^ab^
BUN (mg/dl)	16.04 ± 1.33^a^	15.04 ± 1.47^a^	18.02 ± 2.34^a^	20.63 ± 2.90^a^	18.37 ± 2.09^a^
Crea (mg/dl)	0.58 ± 0.18^a^	0.55 ± 0.15^a^	0.53 ± 0.11^a^	0.53 ± 0.10^a^	0.53 ± 0.14^a^

*Hepatic function*					
AST (U/L)	126.16 ± 3.32^a^	148.53 ± 6.08^b^	122.87 ± 2.03^a^	130.16 ± 0.13^a^	133.64 ± 1.09^a^
ALT (U/L)	43.07 ± 2.42^a^	50.81 ± 3.03^a^	46.09 ± 6.05^a^	43.32 ± 6.08^a^	48.20 ± 3.41^a^
ALP (U/L)	100.19 ± 0.17^ab^	122.06 ± 3.17^c^	104.08 ± 2.13^b^	95.28 ± 2.07^ab^	93.80 ± 3.09^a^

Values are expressed as means ± S.E.M, *n* = 6 rats in each group. There was a significant difference between means that have the different superscript letters in the same row (*p* < 0.05). Statistical analysis was carried out using one-way ANOVA followed by Duncan's test. DM = diabetic rats; Glib = glibenclamide; TG = triglyceride; CHO = cholesterol; LDL = low-density lipoprotein; HDL = high-density lipoprotein; TP = total protein; BUN = blood urea nitrogen; Crea = creatinine; AST = aspartate aminotransferase; ALT = alanine aminotransferase; ALP = alkaline phosphatase.

**Table 5:** Antioxidant activity of PCMOS and PCMAX using DPPH assay, FRAP assay, total phenolic content (TPC), and total flavonoid content (TFC).

Samples	DPPH assay EC_50_ (*µ*g/ml)	FRAP assay (mMFe^2+^/g)	TPC (mgGAE/g)	TFC (mgCE/g)
PCMOS	13.69 ± 0.45^c^	31.23 ± 3.15^a^	5.27 ± 0.31^a^	20.81 ± 0.09^a^
PCMAX	8.03 ± 0.95^b^	42.67 ± 0.77^b^	11.22 ± 0.19^b^	55.08 ± 2.21^b^
Ascorbic acid	0.01 ± 0.02^a^	—	—	—

Values are expressed as means ± S.E.M, *n* = 3 replications. There was a significant difference between means that have the different superscript letters in the same column (*p* ≤ 0.05). Statistical analysis was carried out using one-way ANOVA followed by Duncan's test.

## Data Availability

The data that support the findings of this study are available from the corresponding author upon request.
